# A let-7b binding site SNP in the 3′-UTR of the Bcl-xL gene enhances resistance to 5-fluorouracil and doxorubicin in breast cancer cells

**DOI:** 10.3892/ol.2015.2938

**Published:** 2015-02-06

**Authors:** TING WANG, BIN HUANG, RUI GUO, JIN MA, CUIYING PENG, XUYU ZU, HUIFANG TANG, XIAOYONG LEI

**Affiliations:** 1 Institute of Pharmacy and Pharmacology, University of South China, Hengyang, Hunan 421001, P.R. China; 2 Department of Pharmacy, The Second Affiliated Hospital, University of South China, Hengyang, Hunan 421001, P.R. China; 3 Department of Pharmacy, The First Affiliated Hospital, University of South China, Hengyang, Hunan 421001, P.R. China

**Keywords:** let-7b, B-cell lymphoma-extra large, single nucleotide polymorphism, resistance, breast cancer

## Abstract

The development of acquired resistance to chemotherapy is a major obstacle in the successful treatment of cancer. In breast cancer cells, B-cell lymphoma-extra large (Bcl-xL) is involved in the development of resistance to various chemotherapeutic agents; therefore, preliminary biological prediction was performed to identify a putative binding site for let-7b in the 3′-untranslated region (UTR) of the Bcl-xL gene and a single nucleotide polymorphism (SNP) within this binding region. The present study investigated the association between the SNP rs3208684 A>C and chemotherapeutic agent resistance in breast cancer cells. The data indicated that let-7b negatively regulates the expression of Bcl-xL and appears to sensitize MCF-7 cells to the chemotherapeutic agents 5-fluorouracil (5-FU) and doxorubicin. Furthermore, the SNP rs3208684 A>C was demonstrated to enhance Bcl-xL protein expression by disrupting the binding of let-7b to the 3′-UTR of Bcl-xL and, in MCF-7 cells, overexpression of let-7b in the presence of a mutant Bcl-xL 3′-UTR (C allele) significantly increased 5-FU and doxorubicin resistance. Thus, the results of the present study demonstrate that the SNP rs3208684 A>C may upregulate Bcl-xL protein expression and enhance the resistance of the MCF-7 cells to 5-FU and doxorubicin by decreasing the binding of let-7b to the 3′-UTR of Bcl-xL.

## Introduction

Breast cancer is the leading cause of cancer-related mortality in females, worldwide (1). In the previous two decades, the incidence rate of breast cancer has increased at an average rate of 3.1% per year and the mortality rate has increased at an average rate of 1.8% per year (2). Chemotherapy is an important adjuvant systemic therapeutic approach for the successful treatment of breast cancer (3) and, during early-stage breast cancer, has been demonstrated to improve survival rate (4).

MicroRNAs (miRNAs) have previously been identified as important regulators of a number of key genes associated with chemoresistance (5,6). miRNAs are a class of endogenous, 18 to 25-nucleotide long, non-coding RNAs, which regulate gene expression at the post-transcription level (7,8). As a recognition mechanism, miRNAs complementarily pair to the 3′-untranslated region (UTR) of their target mRNAs, resulting in decreased translational efficiency and/or decreased mRNA expression levels (9–11). It has previously been reported that miRNAs commonly deregulate gene expression levels in specific types of human cancer, and may serve as oncogenes or tumor suppressors (12,13). However, dysregulated miRNAs appear to be associated with every aspect of the cancer-related biological process, including tumor progression, invasion and metastasis, as well as the acquisition of resistance to various chemotherapeutic agents (14,15). Previous studies have indicated that single nucleotide polymorphisms (SNPs) occurring in or near miRNA binding sites may be associated with tumor susceptibility and chemotherapeutic response in humans (6,17–19).

B-cell lymphoma-extra large (Bcl-xL) belongs to the Bcl-2 protein family and appears to confer resistance to apoptosis, thereby reducing the effectiveness of chemotherapy (20). It has previously been reported that overexpression of Bcl-2 and Bcl-xL increases resistance to totaxol and etoposide administration in MCF-7 cells (21), whereas their downregulation sensitizes MCF-7 and MDA-MB-231 cells to doxorubicin, paclitaxel and cyclophosphamide administration (22). In the present study, we hypothesized that SNPs located in let-7b binding sites of the Bcl-xL gene 3′-UTR may regulate Bcl-xL expression, thus, increasing cellular resistance to chemotherapeutic agents in breast cancer cells. To investigate this hypothesis, bioinformatic analyses were performed to identify SNPs in the 3′-UTR of the Bcl-xL gene. We then functionally validated SNP rs3208684 A>C, which is located in the let-7b binding site in the 3′UTR of the Bcl-xL gene.

## Materials and methods

### SNP selection

To predict putative miRNA binding sites in the Bcl-xL 3′-UTR, microrna.org (http://www.microrna.org/microrna/home.do), PicTar (http://pictar.mdc-berlin.de/cgi-bin/PicTar_vertebrate.cgi) and TargetScan version 6.2 (http://www.targetscan.org/) were used. Furthermore, the National Center for Biotechnology Information SNP database (dbSNP; http://www.ncbi.nlm.nih.gov/SNP) was used to identify SNPs within putative miRNA target sites in the 3′-UTR of Bcl-xL. The search was focused on the miRNA seed region, as the seed sequence nucleates interaction between the miRNA and the complementary Bcl-xL mRNA target region, and is the predominant determinant for successful miRNA targeting.

### Cell culture and transfections

The human breast cancer cell line, MCF-7 (Cell Bank of the Chinese Academy of Sciences, Shanghai, China) was cultured in Dulbecco’s modified Eagle medium (HyClone Laboratories, Inc., Logan, UT, USA) with 10% fetal bovine serum (HyClone Laboratories, Inc.) at 37°C and 5% CO_2_. Upon reaching 70% confluence, the MCF-7 cells (5×10^3^ cells per well) were transfected with let-7b mimics or control miRNA mimics [normal control (NC)] (GenePharma Co., Ltd, Shanghai, China), or wild-type Bcl-xL (WT-Bcl-xL), mutant Bcl-xL (Mut-Bcl-xL) or vector alone (pcDNA3.1) (Invitrogen Life Technologies, Shanghai, China) using Lipofectamine^®^ 2000 transfection reagent (Invitrogen Life Technologies, Carlsbad, CA, USA), according to the manufacturer’s instructions.

### Luciferase reporter assay

To conduct the luciferase reporter assay, HEK293T cells (Land Co., Ltd, Guangzhou, China) were seeded onto 24-well plates (2×10^4^ cells per well). In each well, HEK293T cells were transfected with 0.5 μg Bcl-xL 3′-UTR luciferase reporter plasmids containing A or C alleles (Land Co., Ltd) and 100 nM let-7b mimics, let-7b inhibitor or NC inhibitor using Lipofectamine 2000 (Invitrogen Life Technologies), according to the manufacturer’s instructions. At 48 h post-transfection, the cell lysates were collected and a dual luciferase reporter assay system was used to measure firefly and Renilla luciferase activity (Promega Corporation, Madison, WI, USA); relative luciferase activity was calculated by normalizing the Renilla luciferase activity to the firefly luciferase activity.

### Western blot analysis

The cell lysates were separated on 10% SDS-PAGE and transferred to polyvinylidene fluoride membranes (EMD Millipore Co., Hayward, CA, USA). The membranes were blocked with 5% skimmed milk for 1 h and incubated with rabbit monoclonal anti-Bcl-xL (cat. no. 2764), anti-Bcl-2-associated X protein (Bax) (cat. no. 5023) and anti-β-actin (cat. no. 4970) (Cell Signaling Technology, Inc., Danvers, MA, USA) antibodies at dilutions of 1:1,000 overnight at 4°C, respectively. Subsequently, the membranes were incubated with a polyclonal goat anti-rabbit horseradish peroxidase-conjugated secondary antibody (dilution, 1:4,000; cat. no. 7074; Cell Signaling Technology, Inc.) for 1 h. Finally, enhanced chemiluminescence (Enhanced Chemiluminescence Western Blotting kit; Amersham Biosciences, Piscataway, NJ, USA) was used to visualize the results and β-actin was used as internal control.

### 3-(4, 5-dimethylthiazol-2-yl)-2,5-diphenyltetrazolium bromide (MTT) assay

Cell viability was detected by performing an MTT (Sigma Aldrich, St. Louis, MO, USA) assay. MCF-7 cells were transfected prior to treatment with 0, 6, 60, 600, 6,000 and 60,000 μM 5-fluorouracil (5-FU) (Sigma-Aldrich) or 0.0, 0.25, 0.5, 1.0, 2.0 and 4.0 μM doxorubicin (ADM) (Sigma-Aldrich) for 48 h. Following a 4-h re-incubation with 10% MTT solution, 150 μl DMSO (Sigma-Aldrich) was added to solubilize the resultant formazan crystals. The absorbance of the plate was measured in a microplate reader reader (ELX-800, Bio-Tek Instruments, Inc., Winooski, VT, USA) at a wavelength of 570 nm, with a reference wavelength of 650 nm, and the results were expressed as the percentage of absorbance relative to the untreated controls.

### Statistical analysis

Statistical analyses were performed using GraphPad Prism software (version 5.0; GraphPad Software Inc., La Jolla, CA, USA) and Student’s t-test. Data are expressed as the mean ± standard deviation and P<0.05 was considered to indicate a statistically significant difference.

## Results

### Identification of let-7b SNP binding sites in the Bcl-xL 3′-UTR

**Figure 1:**
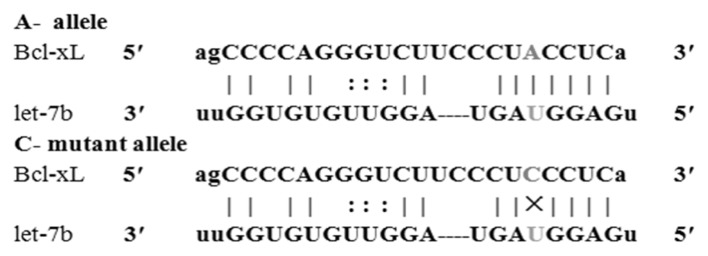
A let-7b SNP binding site in the Bcl-xL 3′-untranslated region. The SNP (rs3208684 A>C) occurs in the 6-bp seed sequence of complementarity at the 5′ end of let-7b. SNP, single nucleotide polymorphism; Bcl-xL, B-cell lymphoma-extra large.

To identify possible miRNA binding sites in the 3′-UTR of the Bcl-xL gene, bioinformatic analysis was performed using three online prediction programs (PicTar, TargetScan and microrna.org). According to the putatively identified miRNA binding sites combined with information from the dbSNP database, it was identified that the Bcl-xL 3′-UTR SNP rs3208684 A>C is located within a predicted miRNA binding site for let-7b (Fig. 1). These results indicate that the C allele forms a non-perfect pairing with the let-7b miRNA seed and, thus, may escape let-7b-mediated regulation.

### Let-7b negatively regulates the protein expression levels of Bcl-xL and sensitizes MCF-7 cells to 5-FU and ADM

**Figure 2:**
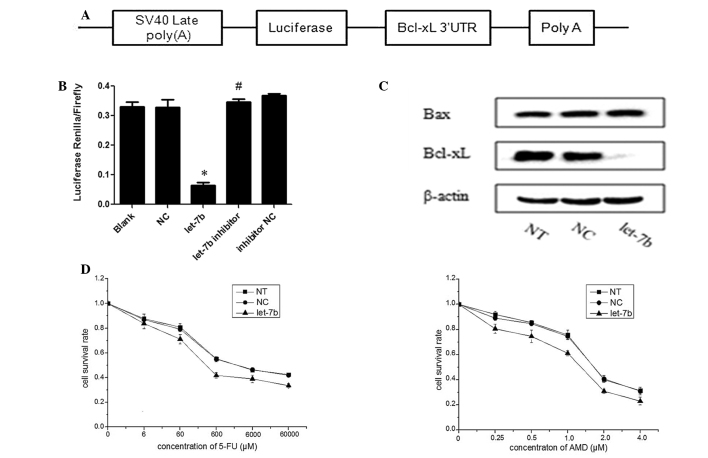
Let-7b negatively regulates the expression of Bcl-xL and sensitizes MCF-7 cells to 5-FU and ADM. (A) Bcl-xL 3′-UTR was cloned into the psiCHECK-2 vector. (B) Luciferase reporter plasmids containing the wild-type Bcl-xL 3′-UTR were transfected in HEK293T cells with 100 nM let-7b, NC, let-7b inhibitor or inhibitor NC and luciferase expression was measured 48 h after transfection. The data are represented as the mean ± standard deviation (SD) of a minimum of three independent transfections analyses. ^*^P<0.01 vs. NC; ^#^P>0.05 vs. inhibitor NC. (C) Let-7b mimics were transfected into MCF-7 cells, and, 48 h after transfection, cells lysates were prepared and subjected to western blot analysis. NT and NC were used as negative controls, and the data are represented as the mean ± SD of three independent analyses. (D) MCF-7 cells were transfected with let-7b mimics or NC and treated with different concentrations of 5-FU or ADM. ^*^P<0.05 vs. NC. The cell survival rate was determined by performing an MTT assay and the data are represented as the mean ± SD of a minimum of three independent transfections analyses. ADM, doxorubicin; Bcl, B-cell lymphoma-extra large; FU, fluorouracil; let-7b, let-7b mimic; MTT, 3-(4, 5-dimethylthiazol-2-yl)-2,5-diphenyltetrazolium bromide; NC, normal control (microRNA mimic); NT, non-transfected cells; SV40 late poly(A), Simian virus 40 late polyadenylation (A); UTR, untranslated region.

Bioinformatic analysis identified a potential binding site of let-7b in the 3′-UTR of the Bcl-xL gene and a dual-luciferase reporter was performed to determine whether let-7b binds at this putative binding site. The full length Bcl-xL 3′-UTR was cloned into the psiCHECK-2 vector (Fig. 2A) and this psiCHECK2-WT-Bcl-xL 3′-UTR vector was subsequently cotransfected into HEK293T cells with let-7b mimics, NC, let-7b inhibitor or inhibitor NC. As indicated in Fig. 2B, luciferase activity was significantly suppressed in the presence of let-7b mimics compared with the NC (P<0.01), whereas the luciferase activity displayed no significant difference in cotransfection rate between the let-7b inhibitor and NC inhibitor groups (P>0.05).

To exert their function, miRNAs suppress the expression of their target genes (23). To verify whether Bcl-xL is a target of the miRNA let-7b, Bcl-xL protein expression levels were analyzed in response to enforced expression of let-7b in MCF-7 cells. Following transfection with let-7b mimics or NC, MCF-7 cells were analyzed by performing western blot analysis and it was determined that overexpression of let-7b significantly inhibited endogenous Bcl-xL protein expression levels. However, overexpression of let-7b had no effect on Bax protein expression levels (Fig. 2C). These biochemical findings indicate that let-7b is a posttranscriptional regulator of Bcl-xL expression in breast cancer cells.

To evaluate the effect of let-7b on the response of MCF-7 cells to 5-FU and ADM treatment, let-7b mimics or NC were transfected into MCF-7 cells and the sensitivity of these mimic-transfected cells to different concentrations of 5-FU or ADM was determined. The MTT assay indicated that MCF-7 cells overexpressing let-7b were significantly more sensitive to 5-FU and ADM, compared with the control cells (P<0.05; Fig. 2D).

### SNP rs3208684 A>C increases Bcl-xL expression levels by interfering with let-7b function

**Figure 3:**
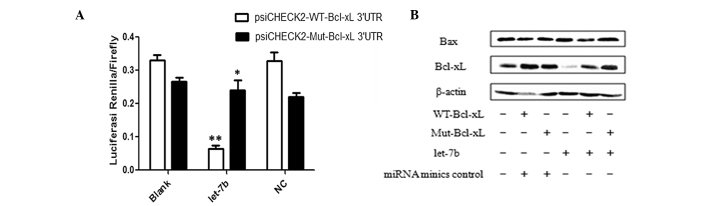
Single nucleotide polymorphism rs3208684 increases Bcl-xL expression by interfering with let-7b function. (A) Luciferase reporter plasmids containing wild-type (psiCHECK2-WT-Bcl-xL-3′-UTR) and mutant (psiCHECK2-Mut-Bcl-xL-3′-UTR) Bcl-xL 3′-UTRs were co-transfected with let-7b mimics or NC into HEK293T cells and luciferase expression was measured 48 h after transfection. The data are represented as the mean ± standard deviation of a minimum of three independent transfections analyses. (B) Bcl-xL gene containing a WT-Bcl-xL or Mut-Bcl-xL 3′-UTR were co-transfected into MCF-7 cells with let-7b. Cell lysates were prepared and subjected to western blot analysis 48 h after transfection. ^*^P>0.05 vs. NC; ^**^P<0.001 vs. NC. Bax, Bcl-2-associated protein X; Bcl, B-cell lymphoma-extra large; miRNA, micro RNA; mut, mutant; NC, normal control (miRNA mimic); UTR, untranslated region; WT wild-type.

As SNP rs3208684 A>C is located within the let-7b seed binding site, we hypothesized that this SNP may result in differential regulation of Bcl-xL induced by let-7b due to the differential binding affinity of let-7b for the two Bcl-xL 3′-UTR genotypes. To investigate this hypothesis, the wild-type (containing the A allele) and mutant (containing the C allele) Bcl-xL 3′-UTRs were cloned into the dual-luciferase psiCHECK-2 reporter vector, and HEK293T cells were co-transfected with let-7b mimics or NC. It was identified that luciferase activity significantly decreased in the presence of psiCHECK2-WT-Bcl-xL 3′-UTR plasmids (P<0.01) but did not change in the presence of psiCHECK2-Mut-Bcl-xL 3′-UTR plasmids (P>0.05). These data indicate that the SNP rs3208684 A>C may affect let-7b binding to the Bcl-xL 3′-UTR (Fig. 3A).

To determine the effect of the SNP rs3208684 A>C on let-7b-mediated regulation of Bcl-xL expression, Bcl-xL gene expression constructs containing WT-Bcl-xL and Mut-Bcl-xL were generated and co-transfected into MCF-7 cells with let-7b mimics. As indicated in Fig. 3B, overexpression of let-7b caused a decrease in Bcl-xL gene expression in the presence of WT-Bcl-xL compared with Mut-Bcl-xL; however, the SNP rs3208684 A>C did not change Bax protein expression levels. Thus, the present study proposes that variation in the SNP rs3208684 A>C may mediate the upregulation of Bcl-xL protein expression by interfering with the binding of let-7b to the 3′-UTR of Bcl-xL in breast cancer cells.

### SNP rs3208684 A>C enhances the resistance of MCF-7 cells to 5-FU and ADM

**Figure 4:**
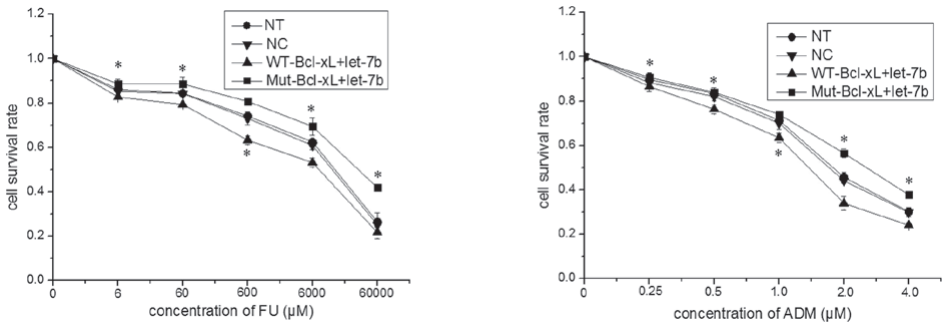
Single nucleotide polymorphism rs3208684 A>C enhances the resistance of MCF-7 cells to 5-FU and ADM. The expression constructs of the full-length Bcl-xL gene containing A (WT-Bcl-xL) or C (Mut-Bcl-xL) were co-transfected with let-7b into MCF-7 cells and, 24 h after transfection, the MCF-7 cells were treated with various concentrations of 5-FU or ADM for 48 h. The cell survival rate was determined by performing an MTT assay and the data are expressed as the mean ± standard deviation of a minimum of three independent transfection analyses. ^*^P<0.05 vs. WT-Bcl-xL+let-7b. ADM, doxorubicin; Bcl, B-cell lymphoma-extra large; FU, fluorouracil; MTT, 3-(4, 5-dimethylthiazol-2-yl)-2,5-diphenyltetrazolium bromide; mut, mutant; NT, non-transfected cells; WT wild-type; NC, normal control (miRNA mimic).

The current study investigated whether the presence of an SNP in the let-7b binding site of the Bcl-xL 3′-UTR results in resistance to chemotherapeutic agents. WT-Bcl-xL or Mut-Bcl-xL were co-transfected into MCF-7 cells with let-7b mimic and, after 24 h, these transfected MCF-7 cells were treated with various concentrations of 5-FU or ADM for an additional 48 h. As indicated in Fig. 4, the survival rate of MCF-7 cells transfected with Mut-Bcl-xL and let-7b was significantly higher than that in MCF-7 cells transfected with WT-Bcl-xL and let-7b (P<0.05). Thus, the SNP rs3208684 A>C in the let-7b binding site of Bcl-xL 3′-UTR appears to result in let-7b-associated 5-FU and ADM resistance in MCF-7 cells.

## Discussion

Bcl-xL is one of two protein products of the Bcl2l1 gene (24) and is a primary antiapoptotic factor, which has been recognized to mediate chemotherapeutic agent resistance. Previously, Bcl-xL and Bax were identified as key factors in the regulation of apoptosis by homodimerization and heterodimerization (25). The human let-7 family is classified as a tumor suppressor family in human cancer (26) and a number of previous studies have indicated that the expression of members of the let-7 family are significantly downregulated in various types of cancer (27), including breast cancer; furthermore, this downregulation is associated with a poorer clinical outcome (28). In the present study, let-7b was demonstrated to target Bcl-xL, resulting in its downregulation in MCF-7 cells. In addition, the present study indicated that let-7b enhances the sensitivity of MCF-7 cells to ADM and 5-FU. These results indicate that let-7b overexpression may enhance cellular sensitivity to 5-FU and ADM via the repression Bcl-xL expression in MCF-7 cells.

In a number of critical genes, SNPs at or adjacent to miRNA binding sites are associated with the chemotherapeutic response of a tumor via the disturbance or obstruction of miRNA binding (29–32). The possible causes of this are the SNPs located in the ‘seed’ regions at the 3′-UTRs of human genes involved in multiple pathways such as cell proliferation, cell death, stress resistance which are likely to affect miRNA-target interaction and target expression accordingly (31). The dual-luciferase reporter assay conducted in the current study revealed that the presence of rs3208684 A-3′-UTR in the let-7b binding site of the Bcl-xL 3′-UTR significantly reduces the expression of luciferase compared with the presence of rs3208684 C-3′-UTR. This is consistent with the initial bioinformatic analysis, which indicated a functional interaction between let-7b and Bcl-xL mRNA. Additionally, the transfection of MCF-7 cells with rs3208684 A-3′-UTR and let-7b mimic demonstrated significant inhibition of Bcl-xL expression, and the SNP rs3208684 A>C was identified to cause 5-FU and ADM resistance in MCF-7 cells. Thus, these results indicate that the occurrence of an SNP in rs3208684 A-3′-UTR of Bcl-xL may contribute to the alteration of cellular resistance to 5-FU and ADM.

In conclusion, the present study, demonstrated that let-7b may enhance the sensitivity of MCF-7 cells to 5-FU and ADM by regulating Bcl-xL expression. The SNP rs3208684 A to C may inhibit the interaction between let-7b and Bcl-xL 3′-UTR, resulting in higher Bcl-xL expression, as well as cellular resistance to 5-FU and ADM. Thus, we propose that the SNP rs3208684 A>C may be a potential marker for personalized therapeutic approaches. Furthermore, these results provide insight into a potential novel chemotherapeutic strategy for breast cancer by combining let-7b with currently used chemotherapeutic agents.
